# Effect of medical education on European primary care physicians’ knowledge in management of major depressive disorder and psychiatric emergencies

**DOI:** 10.1192/j.eurpsy.2022.747

**Published:** 2022-09-01

**Authors:** L. Thevathasan, L. Fairley, C. Phillips

**Affiliations:** Medscape LLC, Clinical Strategy, London, United Kingdom

**Keywords:** Suicide, major depressive disorder, MDD, Psychiatric emergencies

## Abstract

**Introduction:**

The challenge for primary care physicians (PCPs) is keeping up to date in managing major depressive disorder (MDD) and psychiatric emergencies.

**Objectives:**

We evaluated whether an online educational video lecture directed at PCPs, could improve knowledge and confidence regarding management of psychiatric emergencies associated with MDD.

**Methods:**

Educational effect was assessed using a 3-question repeated pairs, pre/post assessment survey. A paired-samples t-test was conducted to assess overall number correct and confidence change. A McNemar’s test was conducted to assess question-level significance. P values < 0.05 are statistically significant. Cohen’s d test was used to estimate the magnitude of effect of education. The activity launched on 8 April 2021, and preliminary data analysed as of 24 June 2021.

**Results:**

511 PCPs participated in the programme, of which 86 PCPs completed the pre- and post-assessment test. An average overall correct response rate of 28% pre- increased to 64% post- (129% relative increase, P<0.001; Cohen’s d = 1.13). Knowledge on the burden of suicide and MDD improved from 23% pre- to 87% post- (278% relative increase,P<0.001). Knowledge regarding clinical data for novel therapies for use in psychiatric emergencies improved from 29% pre- to 50% post- (72% relative increase, P<0.01). Knowledge regarding signs of suicidal intent in patients with MDD improved from 31% pre- to 53% (71% relative increase, P<0.001) following education.

**Conclusions:**

This study demonstrates the positive effect of online medical education on PCPs’ knowledge and confidence in contemporary management of psychiatric emergencies associated with MDD in Europe.

**Disclosure:**

The results of this study were derived from an educational programme which was developed through independent educational funding from Janssen Neuroscience

